# Towards an Alternative to Time of Flight Diffraction Using Instantaneous Phase Coherence Imaging for Characterization of Crack-Like Defects

**DOI:** 10.3390/s21030730

**Published:** 2021-01-22

**Authors:** Baptiste Gauthier, Guillaume Painchaud-April, Alain Le Duff, Pierre Bélanger

**Affiliations:** 1 PULÉTS, École de Technologie Supérieure (ÉTS), Montréal, QC H3C 1K3, Canada; pierre.belanger@etsmtl.ca; 2 Olympus NDT Canada, Québec, QC G1P 0B3, Canada; guillaume.painchaud-april@olympus.com (G.P.-A.); alain.leduff@olympus.com (A.L.D.)

**Keywords:** ultrasonic phased array, TOFD, ultrasonic imaging, TFM, instantaneous phase

## Abstract

Time of flight diffraction (TOFD) is considered a reliable non-destructive testing method for the inspection of welds using a pair of single-element probes. On the other hand, ultrasonic phased array imaging has been continuously developed over the last couple of decades, and now features powerful algorithms, such as the total focusing method (TFM) and its multi-view approach to rendering detailed images of inspected parts. This article focuses on a different implementation of the TFM algorithm, relying on the coherent summation of the instantaneous signal phase. This approach presents a wide range of benefits, such as removing the need for calibration, and is highly sensitive to defect tips. This study compares the sizing and localization capabilities of the proposed method with the well-known TOFD. Both instantaneous phase algorithm and TOFD do not take advantage of the signal amplitude. Experimental tests were performed on a ¾″-thick steel sample with crack-like defects at different angles. Phase-based imaging techniques showed similar characterization capabilities as the standard TOFD method. However, the proposed method adds the benefit of generating an easy-to-interpret image that can help in localizing the defect. These results pave the way for a new characterization approach, especially in the field of automated ultrasonic testing (AUT).

## 1. Introduction

Welds are used in a wide range of industries to assemble metal parts, such as pipes and plates. Their integrity is crucial to providing the safe and efficient operation of safety-critical components [1]. Whether originating from the manufacturing process or in-service operations, defects can lead to dramatic failures if not detected at an early stage. Cracks, porosities, and lack of fusion are among the common discontinuities that have been observed in welds. Ultrasonic inspection techniques (UT) are among the most common methods used to identify and characterize such defects. For the inspection of girth welds, the typical inspection setup comprises conventional ultrasonic phased array (PA) probes and additional time of flight diffraction (TOFD) transducers operating at higher frequencies [2].

Introduced in the early 1970s for nuclear industry vessels, TOFD relies on a pitch-catch configuration, in which diffracted ultrasonic energy allows accurate sizing of defects. However, this technique presents some limitations, notably related to the interpretation and localization of indications, important dead zones, and a strong signal attenuation [3]. On the other hand, conventional phased array ultrasonic testing methods, which have long been used for linear or raster scanning, are also very interesting for their electronic scanning capabilities. Indeed, such techniques allow almost any type of acquisition scheme to steer, focus, or sub-aperture the ultrasonic beam that is sent to the sample. Particularly, the full matrix capture (FMC) [4,5] data acquisition scheme, and its total focusing post-processing method (TFM) [6,7] have proven to be reliable, and provide easy-to-understand images, while using conventional probing hardware [8,9].

Recently, the multi-view (or multi-mode) technique, using various combinations of ray path, backwall reflections, and mode conversions, has been explored [10,11,12]. This technique mimics virtual probes, which should allow imaging of the same defect from different points of views. However, it also comes with some drawbacks, as the large number of views that can be considered can rapidly increase the complexity. Thus, at the time of writing, the selection of the relevant modes to image a given defect is highly reliant on the inspector’s experience. Among the different strategies that are currently being developed to overcome such complexity, sensitivity maps [13,14,15] and fusion of views [16] are very promising, but even they still rely on known defect characteristics.

While conventional TFM consists of the summation of time domain amplitude signals, the phase of signals can however also be fed directly to delay and sum algorithms for image reconstruction, or as a weighting factor [17]. Additionally, since defect sizing currently relies on calibrated amplitude, direct phase-based image reconstruction allows a different approach, in which calibration is no longer a requirement, as amplitude information is simply disregarded. It appears that in the case of narrow defects, tips are particularly well represented in such images, which allow accurate sizing and localization of reflectors [18].

In this paper, instantaneous phase coherence imaging (IPCI) and conventional TFM are compared to TOFD. This comparison was motivated by the fact that both the IPCI and TOFD methods are not based on amplitude to detect and size indications. Performance, in terms of sizing and localization of notch-like defects, was compared and assessed on a low-carbon steel plate. The rest of the paper is divided as follows: Section 2 presents the materials and methods, including: a description of TOFD and its limitations, the instantaneous coherence imaging algorithm, as well as the experimental setup. Experimental results from different methods are depicted in Section 3, followed by a discussion in Section 4. Finally, conclusions are presented in Section 5.

## 2. Materials and Methods

### 2.1. Time of Flight Diffraction and Its Limitations

**Figure 1:**
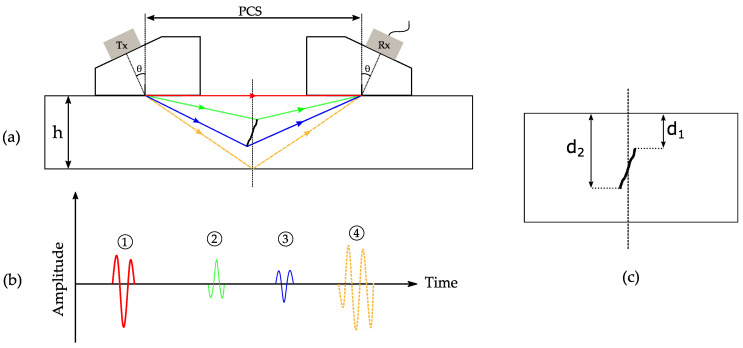
Time of flight diffraction principle: (**a**) setup constituted by two probes in opposition, mounted on a wedge of angle *θ*. The probes are separated by a distance, PCS (probe center spacing), and placed on a sample of thickness, h; (**b**) time traces as recorded by the receiving transducers displaying the lateral wave (1), the upper diffraction response (2), the lower tip diffraction response (3), and the backwall echo (4); (**c**) measurement of the depth and height of a defect.

In this section, TOFD is briefly introduced, and known limitations of the method are presented and discussed. As illustrated in Figure 1a, TOFD commonly relies on the use of two single-element probes on opposite sides of a region of interest acting in a pitch-catch configuration, with emitter Tx and receiver Rx. The distance between the center of the probes, s, also called PCS (probe center spacing), can be calculated by:

(1) s=2×d×tan(θ)

where d is the depth of focus, and θ the angle of the wedge. For samples of thickness, h, up to 50 mm, θ is usually equal to 60° or 70°, and d is defined by:

(2) $$d=\frac{2}{3}\times h$$

for adequate coverage [19]. Taking the example of a defect which has two extremities not breaking to the surface, TOFD is characterized by four well defined echoes (Figure 1b). First, the lateral wave (similar to creeping waves, a wave originating at the upper part of the envelop of a compression wave for which propagation occurs at a depth of a few millimeters and parallel to the surface) is recorded, and has the shortest time of flight. If it is not recorded, or received with a weak amplitude, then that is a good indication of a surface crack. Additionally, the lateral wave is often used for assessing coupling conditions and for calibration purposes. Then, when the ultrasonic beam encounters an indication along its path, typically a crack, a part of the energy will be reflected and the remainder diffracted from the tips of the indication, which are then acting like point sources emitting a spherical wavefront. This diffracted pattern will be recorded by the receiving probe, and the upper and lower tip will have a characteristic 180-degree phase shift. Lastly, the reflection of the backwall, with a high amplitude, is received.

#### 2.1.1. TOFD Defect Sizing and Localization

The sizing (height) and localization (depth) of the defect can be estimated by measuring the time delay for the signals shown in Figure 1b, and using the knowledge of the sample longitudinal velocity, the wedge delay, and the PCS. Thus, considering an isotropic material, a flaw tip depth, $d_{i}$, is given by [20]:

(3) $$d_{i}=\;\sqrt{{\left(\frac{c\;\left(t-2t_{0}\right)}{2}\right)}^{2}-{\left(\frac{s}{2}\right)}^{2}}$$

with c being the longitudinal velocity in the sample material, t the total time from the transmitter to the receiver for the flaw tip indication, and $t_{0}$ the propagation time in the wedge material. The flaw height, b, can then be expressed as:

(4) $$b=\;d_{2}\;-\;d_{1}$$

where $d_{1}$ is the upper tip depth, and $d_{2}$ the lower tip depth, as illustrated in Figure 1c. Here, a conventionally adopted assumption is made, that the defect lies exactly midway between the transducers [21]. Additionally, a phase shift of π is observed between the upper and lower tips of the crack [22], enabling easy identification on time traces.

#### 2.1.2. TOFD Limitations

Notwithstanding the apparent simplicity of this method and the fact that sizing does not rely on signal amplitude, TOFD presents several limitations that may affect the detectability, sizing, and localization of discontinuities.

##### Dead Zones and Spatial Resolution

**Figure 2:**
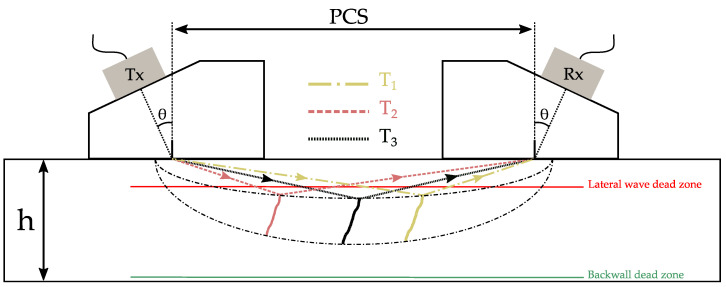
Dead zones of time of flight diffraction (TOFD) related to the ring time near the surface (lateral wave dead zone) and the backwall (backwall dead zone). Locus curve (dotted line) showing three possible defect locations for the exact same times of flight ($T_{1}$, $T_{2}$, and $T_{3}$).

There are two dead zones (Figure 2) related to the ring time, near the surface and the backwall. They can be easily evaluated considering the exciting pulse duration, PCS, and material velocity [23]:

- The lateral wave dead zone limits the detection capabilities directly beneath the surface of the sample. This dead zone usually has a significant depth of 4 to 8 mm (for frequencies in the 5 MHz–15 MHz range and steel samples of thickness below 25 mm) that can be limited by reducing PCS and/or reducing the pulse duration (i.e., using a higher frequency on a highly damped probe).
- The backwall dead zone limits the detection capabilities directly above the backwall of the sample. The thickness of this dead zone is usually less than 1 mm, and must therefore be considered when looking for small defects in this area.

Additionally, particular attention should be given to spatial resolution, which decreases with decreasing depth [24]. Thus, the shallowest defect for detection has to be considered when evaluating the acceptability of the method. However, the spatial resolution can be increased by increasing the frequency of the signal or reducing the PCS or pulse width.

##### Locus Curve

As mentioned earlier with respect to the localization and sizing of the defect, it is assumed that the defect lies in the middle of the two probes. Additionally, the measurement carried out relies solely on the arrival time of the different echoes. However, and for a given pair of transducers, several defect locations and sizes exist for the exact same recorded time traces. As illustrated in Figure 2, if the center of the probes is considered as the foci of an ellipse, then the dotted line represents the locus of all points having the same travel time, $T_{1}\;=\;T_{2}\;=\;T_{3}$ [3]. Thus, if the defect is not perfectly at the center of the two probes, errors can be made when estimating its depth and height.

### 2.2. Instantaneous Phase Coherence Imaging

**Figure 3:**
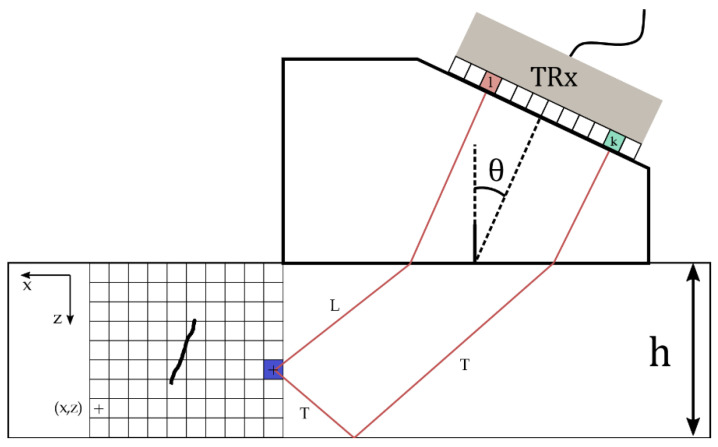
Data acquisition principle through full matrix capture (FMC): a signal is emitted by element *i* and received by element *j*. Image reconstruction area is divided into pixels, each one denoted by its position (*x*,*z*).

A different approach, which also allows sizing and localization of defects through an imaging technique, and having the potential to become an alternative solution to traditional TOFD, is presented in this section. Instantaneous phase coherence imaging uses the complete set of signals (FMC) recorded from each pair of emitting elements, i, and receiving elements,  j, of a 1-D linear array (Figure 3). After data is acquired, each signal is delayed and summed according to the distances between the emitter, the pixel, and the receiver. Finally, the result is mapped to the image domain. This imaging method, known as the total focusing method (TFM) [4], has traditionally relied on the summation of the delayed amplitude of signals. However, the amplitude is affected by numerous phenomena along the wave path, such as diffraction at the probe level, free propagation (i.e., geometric and inhomogeneities scattering losses), interaction with interfaces (i.e., transmission and reflection coefficients, mode conversions), and scattering by the flaw. Although the amplitude correction associated with these factors could be factored out by an appropriate model, the computational complexity may lead to performance issues. Currently, this issue is alleviated by acquiring empirical amplitudes obtained on representative flaws. The complete process, referred to as amplitude calibration, is time consuming and prone to statistical fluctuations (e.g., generically, the acquisition circumstances of the calibration step may differ from the actual inspection step). It is therefore desirable to obtain an imaging process that is less impacted by amplitude-based aberrations. Thus, in this section, an algorithm derived from TFM is presented; it relies only on the coherent summation of signal instantaneous phase [17]. This algorithm has been considered in the past as a weighting factor for amplitude-based images in order to reduce artefacts [18].

Multi-view imaging is also explored as it has recently proven to be an interesting technique for defect characterization [10,12,25]. A view is constituted of two ray paths: a transmitting path from the probe to the defect, and a receiving path from the defect to the probe. Each ray path can be composed of both the longitudinal (L) and transverse (T) wave modes, with or without reflection from the backwall. As the first and final legs of the ray path are in the wedge, which is only used for longitudinal wave propagation, they are omitted in the nomenclature [26]. For example, the TT-L path is made of a TT half-skip path (transmission) from the probe to the defect, and an L direct path (reception) from the defect to the probe.

**Figure 4:**
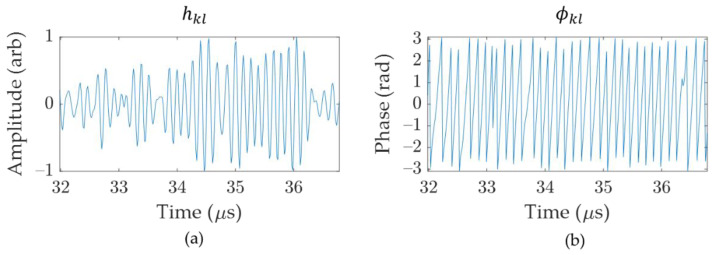
Sample time traces for: (**a**) amplitude signal, $h_{kl}$, and (**b**) instantaneous phase, $\phi_{kl}$.

Considering a 1-D linear array of N elements, the received signal at element l from an emission at element k is denoted $h_{kl}\left(t\right)$ (Figure 4a). For a conventional TFM, every pixel intensity, $I_{TFM}$, of the image located at position (x,z)  is obtained by summing the delayed amplitude of the signal [4]:

(5) $$I_{TFM}\left(x,z\right)=\left|\overset{N}{\underset{k=1}{\sum }}\overset{N}{\underset{l=1}{\sum }}{\hat{h}}_{kl}(\tau_{kl}\left(x,z\right))\right|=\left|\overset{N}{\underset{k=1}{\sum }}\overset{N}{\underset{l=1}{\sum }}\left|{\hat{h}}_{kl}(\tau_{kl}\left(x,z\right)\right|e^{i\phi_{kl}\left(\tau_{kl}\left(x,z\right)\right)}\;\right|$$

with ${\hat{h}}_{kl}\left(t\right)$ being the Hilbert transform of $h_{kl}\left(t\right)$, and $\tau_{kl}\left(x,z\right)$ the total time of flight from the transmitting to the receiving element for the considered view. In the case of IPCI, every pixel intensity, $I_{IPCI}$, of the image located at position (x,z)  is obtained by summing the delayed in-phase and quadrature components of signals [27]:

(6) $$I_{IPCI}\left(x,z\right)=\frac{1}{N^{2}}{\left({\left(\overset{N}{\underset{k=1}{\sum }}\overset{N}{\underset{l=1}{\sum }}\sin(\phi_{kl}\left(\tau_{kl}\left(x,z\right)\right)\right)}^{2}+{\left(\overset{N}{\underset{k=1}{\sum }}\overset{N}{\underset{l=1}{\sum }}\cos(\phi_{kl}\left(\tau_{kl}\left(x,z\right)\right)\right)}^{2}\right)}^{m}\;$$

with $\phi_{kl}$ being the instantaneous phase of the signal (Figure 4b) defined by:

(7) $$\phi_{kl}\left(t_{kl}\right)=\arctan\left(\frac{{\hat{h}}_{kl}\left(t_{kl}\right)}{h_{kl}\left(t_{kl}\right)}\right)$$

Exponent m in Equation (6) is used as a modulation factor to increase or decrease the sensitivity to side lobes and reduce the main lobe. In this paper, the typical value of m=0.5 is used, which corresponds to the envelope of the instantaneous phase. Thus, Equation (6) can be rewritten in a simpler form as:

(8) $$I_{IPCI}\left(x,z\right)=\;\left|\overset{N}{\underset{i=k}{\sum }}\overset{N}{\underset{j=l}{\sum }}e^{i\phi_{kl}\left(\tau_{kl}\left(x,z\right)\right)}\;\right|$$

The underlying principle supporting IPCI is the coherence of the phase when adequately delayed signals are summed. The scattered wave resulting from the interaction with a flaw propagates back and is received by each element of the probe. Compared to the phase resulting from the backscattering from a continuous distribution of random minor inhomogeneities in the material, it is assumed that a single major inhomogeneity, a flaw for instance, will produce a more coherent phase structure over the detection array. Hence, the summation of a large number of appropriately delayed coherent signals would result in a large response, in comparison to the summation of many random responses from lesser material inhomogeneities. Interesting properties can then be deduced from the principle of IPCI. The following subsections present three of them, considering only the L–L view for convenience and brevity.

#### 2.2.1. Noise Level Sensitivity

**Figure 5:**
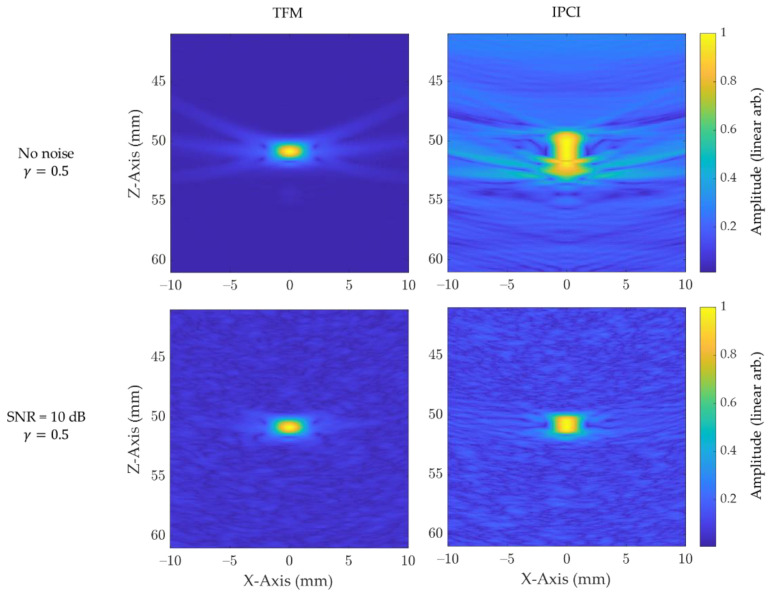
Simulated noise sensitivity for a point like scatterer, using both instantaneous phase coherence imaging (IPCI) and the conventional total focusing method (TFM). The upper row images were generated using raw time traces, while the lower row images where generated with a signal-to-noise ratio (SNR) of 10 dB. A gamma correction factor of 0.5 was applied to all images.

**Figure 6:**
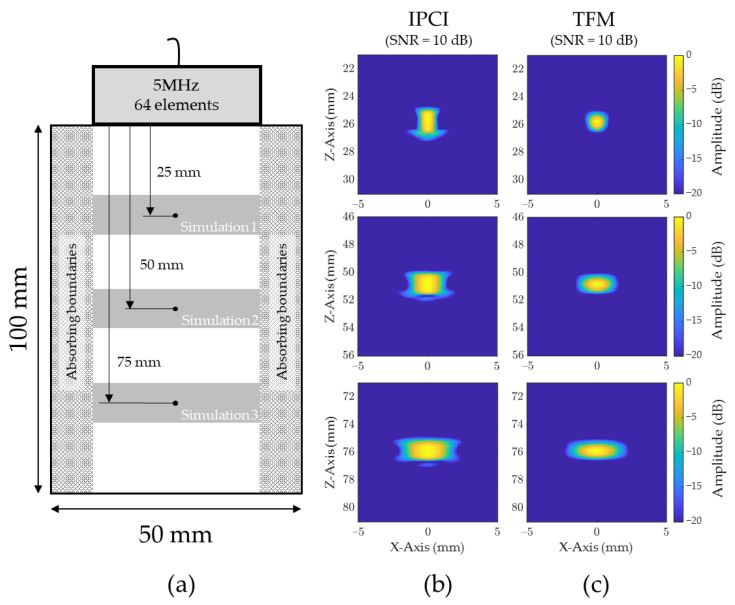
Simulated noise level sensitivity analysis principle for a point like scatterer, using both IPCI and conventional TFM: (**a**) simulation setup showing the 3 different simulations that were conducted, modeling a 5 MHz, 64 element probes and 0.4 mm diameter side drilled holes; (**b**) images obtained for each scatterer, and each simulation using IPCI and an added band-limited Gaussian noise with a SNR of 10 dB; (**c**) images obtained for each scatterer, and each simulation using TFM and an added 10 dB Gaussian noise.

Unlike conventional TFM, IPCI is conditional on the existence of a certain level of noise in the input signals. Indeed, if received signals are composed of perfectly equal or zero valued parts, those parts will be coherent, and hence produce artefacts in the final image (Figure 5). Additionally, the restitution of a given scatterer will also be affected by this noise level. To illustrate this behavior, three simple finite element simulations were conducted, modeling a 5 MHz (64 element) 0.5 mm pitch probe positioned above a low-carbon steel sample of dimensions 100 mm × 50 mm, as illustrated in Figure 6a. The three simulations successively modeled a side drilled hole (SDH) of a diameter of 0.4 mm located at different depths (simulation 1:25 mm; simulation 2:50 mm; and simulation 3:75 mm). For each simulation, a 3 cycle Hann windowed toneburst, centred at 5 MHz, was transmitted, and the FMC was recorded and then used as input data for IPCI and TFM. Absorbing boundaries were added to the sample sides so as to prevent unwanted reflections and associated artefacts. Simulations were carried out using the Pogo FEA solver [28], which allows fast computing of such configurations thanks to its GPU based architecture.

In reality, noise can originate from the acquisition chain, the properties of the material under inspection, or both [8]. Usually, electronic noise is alleviated by averaging over time, while the material noise is difficult to attenuate. Gaussian noise, with a signal-to-noise ratio (SNR) of either 5 dB, 10 dB, 15 dB, or 20 dB, was added to raw time traces during post processing for the purpose of this demonstration. This noise, as well as the raw time traces, were also filtered using a bandpass Butterworth filter between 2.5 MHz (half of the transducer central frequency) and 10 MHz (twice the transducer central frequency), so as to reflect a realistic inspection, and in accordance with the new ISO 23865 standard for TFM inspection. Figure 5 illustrates the influence of noise on the reconstructed images for both TFM and IPCI. For both algorithms, the image of the central scatterer (simulation 1) was computed for a noiseless case and for a SNR of 10 dB. A gamma correction factor of 0.5 was applied to images, and a linear scale was used to help the visualization by the reader. For IPCI, the noiseless case shows, as expected, a high level of background noise, due to the high level of coherence outside of the scatterer, while TFM remains lightly affected; many elementary time series with 0 amplitude on all noiseless elementary time series produced large summation values when Equation (8) was applied.

Figure 6b,c shows the reconstructed images (both IPCI and TFM) for the three simulations and a 10 dB SNR. A similar behavior was observed, as the geometrical spreading tends to increase the horizontal extent of indications with increasing depth in both IPCI and TFM. Additionally, the vertical extent of the indications was smaller for TFM than IPCI. However, restitution of the scatterer differed slightly when changing the noise level to other values. Thus, the array performance indicator (API) [4] was calculated for all noise levels and simulations. Table 1 depicts that, with increasing SNR, the API tended to increase when using IPCI, while remaining at acceptable levels. On the other hand, the API was almost unchanged for any noise level when using TFM.

Given that the noiseless case would only be encountered in a simulated inspection, IPCI qualifies for most experimental studies, as a minimal noise level will always be present.

#### 2.2.2. Attenuation Robustness

**Figure 7:**
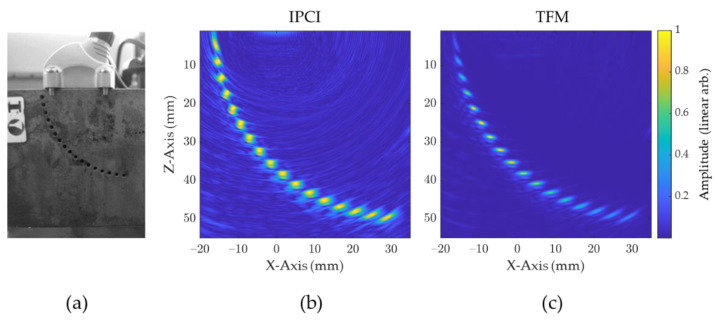
Experimental example of reconstructed defect consistency and attenuation robustness, using IPCI instead of TFM: (**a**) inspection setup composed of a 5 MHz (32 element) probe and an ASTM E2491 low-carbon steel test bloc; (**b**) reconstructed image of the sample obtained using IPCI; (**c**) reconstructed image of the sample obtained using TFM.

IPCI is by definition an amplitude free imaging method. Indeed, the coherent summation of signals happens independently of the amplitude of the signals, and hence produces indications with a high contrast level. This is particularly useful when the appropriate gain to be used for ensuring the detection of a given flaw is unknown. To illustrate this property, an experimental study was conducted in which a 5 MHz (32 element) 1 mm pitch probe was positioned above an ASTM E2491 low-carbon steel test bloc (Figure 7a). The FMC was acquired and then used to feed IPCI and TFM algorithms. From Figure 7b,c it can clearly be seen that all SDH are displayed with the same amplitude for IPCI, whereas the effect of attenuation can be perceived for TFM on the lower SDH, and the effect of the beam transmitted amplitude on the upper SDH. To allow visualization of the whole information of the reconstructed image and to help comparison, a linear scale was used in Figure 7b,c.

#### 2.2.3. Defect Tips Sensitivity

**Figure 8:**
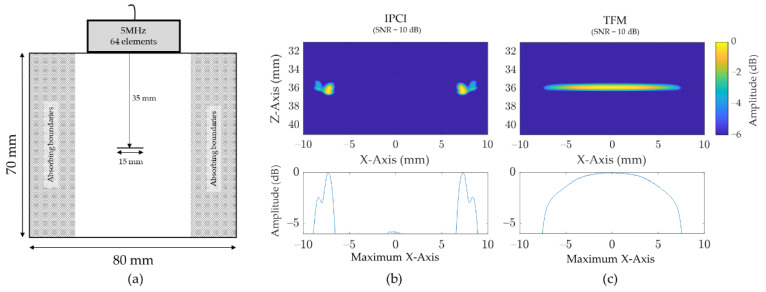
Simulated tips sensitivity analysis on a thin horizontal, using both IPCI and conventional TFM: (**a**) simulation setup showing the modeled notch (0.1 mm thick and 15 mm width) as long as the 5 MHz (64 element) probe above it; (**b**) image obtained using IPCI and added band-limited Gaussian noise with a SNR of 10 dB, as well as its maximum intensity according to *X*-axis; (**c**) image obtained using TFM and added band-limited Gaussian noise with a SNR of 10 dB, as well as its maximum intensity according to *X*-axis.

Another interesting property of IPCI is its sensitivity to defect tips, which allows accurate sizing of discontinuities. Indeed, it appears that the specular reflections coming from large scatterers are reduced with respect to amplitude summation, as only a few common phase terms actually contribute to the summation Equation (8). These few common phase terms do, however possess large amplitude, hence the strong signal from specular reflection in amplitude Equation (5). IPCI, then, tends to be more robust for narrow and point-like reflectors. The same simulation and processing scheme as in Section 2.2.1 was used, modeling the same probe above a 70 mm × 80 mm sample. A thin horizontal notch (0.1 mm thick and 15 mm wide) was modeled, and was located at the center of the bloc (Figure 8a). Again, the FMC was collected, and fed to IPCI and TFM algorithms. Band-limited Gaussian noise (10 dB SNR) was added to the time traces in a similar fashion to the simulation in Section 2.2.1. Images displayed in Figure 8b,c were scaled on a −6 dB range, so as to apply the usual 6 dB drop sizing technique for such defects [29]. Using TFM and the aforementioned sizing method, the crack width was estimated to be 15.1 mm (Figure 8c). On the other hand, using IPCI and the distance between the two tips of the notch in Figure 8b, the crack width was estimated to be 14.7 mm.

Thus, even if in this case TFM gives a more accurate evaluation of the exact extent of the notch, IPCI, thanks to its important sensitivity to defect tips, allows accurate sizing of notch-like defects without using semi-empirical considerations that could be easily misled by a calibration process or attenuation in the material.

### 2.3. Experimental Setup

**Figure 9:**
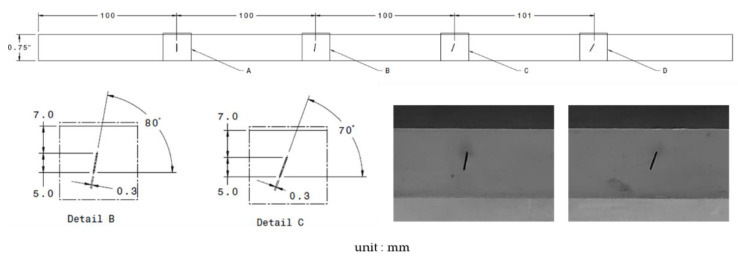
The ¾″ thick low-carbon steel sample plate. Four angled notches of 0.3 mm width were grooved using electrical discharge machining (EDM). Details (left) and pictures (right) are for a 70° (notch C) and 80° (notch B).

The experimental study aimed to assess and compare the performances of both methods (TOFD and IPCI) as reflectors that would be close to what can be encountered in a typical weld inspection process. Thus, experiments were conducted on a medium low-carbon steel 19 mm sample plate within which angled notches of 0.3 mm width were machined using electrical discharge machining (EDM). Notches were centered in the middle of the plate thickness and four different angles were investigated: 90° (notch A), 80° (notch B), 70° (notch C), and 60° (notch D) respective to the horizontal plane (Figure 9). Each notch had the same vertical extent of 5 mm, and the same center position at 9.5 mm from the top or bottom of the plate.

A Vantage 64 LE (Verasonics Inc., Kirkland, WA, USA) phased array acquisition system was used to record FMC for IPCI. TOFD measurements were performed on the commercial phased array platform, Omniscan X3 (Olympus Canada Inc., Quebec, QC, Canada). Single-element transducers used for TOFD had a center frequency of 15 MHz, an element size of 3 mm, and were excited by a square negative pulse. The PCS was set to 69.8 mm according to (2). The 60 element 1-D linear array transducer used for IPCI had a center frequency of 7.5 MHz, an elementary pitch of 1.0 mm, and an element width of 10 mm. A 3-cycle sinusoidal Hann-windowed signal was sent to each element for excitation. The full experiment parameters are listed in Table 2.

**Figure 10:**
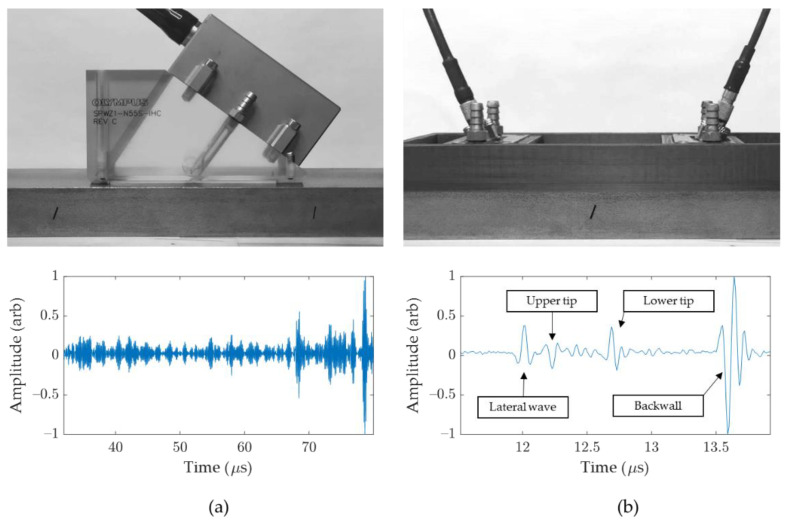
Measurement setup, and typical signal acquired signal for IPCI (**a**) and TOFD (**b**).

The linear array was placed on the right side of the notch, as shown in Figure 10a, mimicking the inspection setup that would be used to scan a weld bead and potential defects that could arise on the weld bevel. A 3D printed frame was used to hold both TOFD probes, and to adjust the PCS. Again, they were place on each side of the notch, as would be done for an automated weld inspection (Figure 10b). Signals were processed in MATLAB R2019b (MathWorks, Natick, MA, USA), and sample time traces are shown in Figure 10a,b for each method. For convenience, signals were time-delayed to remove the excitation signal. It can be noted that the acquisition time was much longer for IPCI, due to the time of flight in the wedge, as well as the multiple reflections to consider for multi-view imaging. However, once a specific view and reconstruction area were defined, a useful time sample could be extracted, and the overall required data considerably reduced. While similar results can easily be obtained with small variations of the linear array position for IPCI, TOFD is very sensitive to PCS [30], the position of the probes relative to the defect, and the coupling quality.

## 3. Results

First, results obtained for the TOFD characterization of notches A to D are detailed in Table 3. The measured height, as well as the defect center position, were calculated according to (3) and (4), and compared to expected values. TOFD performances in terms of localization were very satisfactory, with errors below 5%, and sizing showed a good agreement with errors, below 10%.

**Figure 11:**
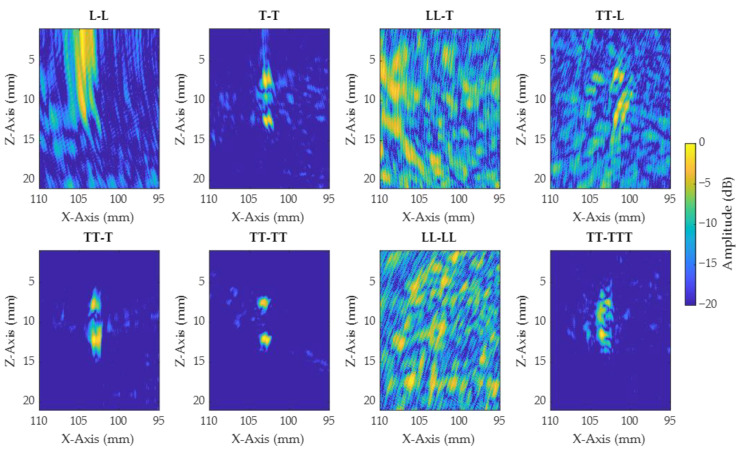
IPCI imaging algorithm applied to different views for notch A. The *X*-axis corresponds to the lateral distance from the wedge and the *Z*-axis to the vertical distance in the sample.

**Figure 12:**
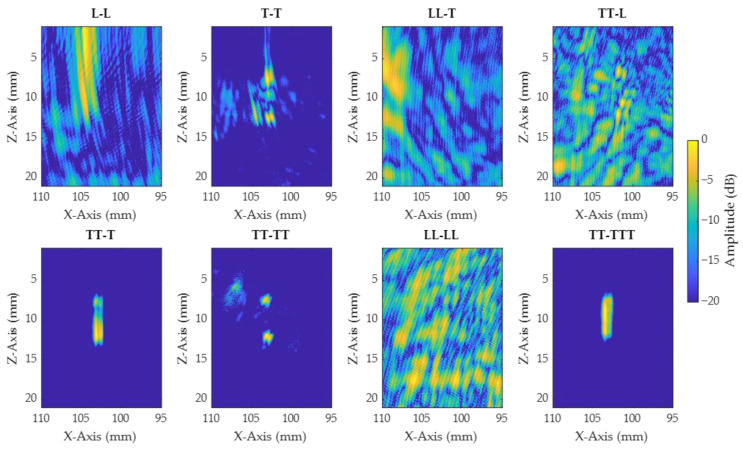
TFM algorithm applied to different views for notch A. The *X*-axis corresponds to the lateral distance from the wedge and the *Z*-axis to the vertical distance in the sample.

In order to compare the TOFD results with IPCI, the best view was first selected. Several views considering direct, half-skip, and full-skip paths were generated to help the selection, and are presented in Figure 11 for notch A. Additionally, conventional TFM images are also presented and used for characterization, in order to give the reader an element of comparison with a well-adopted method (Figure 12). It can be seen that L-based views had low sensitivity to the notches, and consisted mainly of strong artefacts, for both IPCI and TFM. All views composed only of transversal waves denoted a partial, or very clear, reconstruction of the defect. Comparing Figure 11 to Figure 12, we find fewer artefacts and a higher sensitivity to defect tips using IPCI in the former. The same observations were made for images relative to notches B, C, and D. To reduce the number of views considered in the present paper, only views T–T and TT–TT were kept for the rest of the comparison. T–T had a lower acquisition time, while allowing sizing and localization for most cases. Additionally, TT–TT was the better view for the characterization of the notches, but had a longer acquisition time.

**Figure 13:**
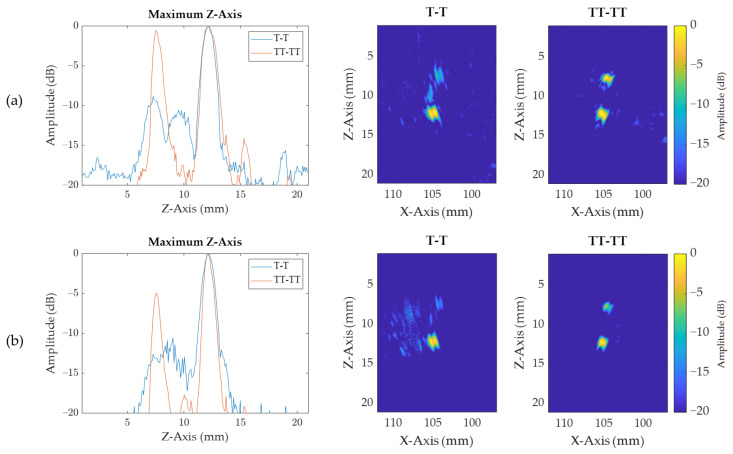
T–T and TT–TT views of notch B and their maximum intensity according to *Z*-axis for (**a**) IPCI and (**b**) TFM.

Once T–T and TT–TT views were generated for each notch and each imaging algorithm (TFM and IPCI), the maximum intensity according to the *Z*-axis could be plotted to allow easy characterization of the defect in terms of position and sizing, as shown in Figure 13 for notch B. Both Z positions of local maxima corresponding to crack tips were measured to obtain the height and the center depth of each notch, as detailed in Table 4 and Table 5. Additionally, using the TT–TT view and a −10 dB threshold, the angle of the defect could be determined using the principal axis, determined through calculation of the moment of the image [31].

**Figure 14:**
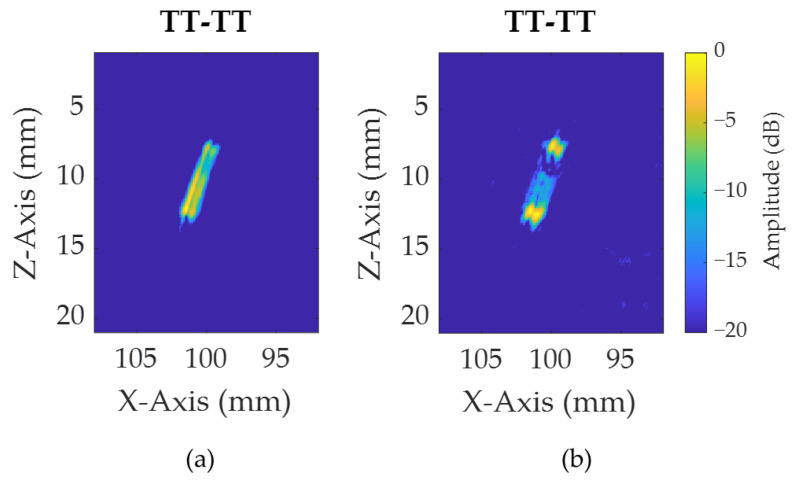
TT–TT views of notch C for (**a**) TFM and (**b**) IPCI.

For the results displayed in Table 4 and Table 5, some values are in brackets, as they can be discarded because of the poor ability of the reconstructed image to allow the identification of defect tips. This is mainly observed for notches having an angle (Figure 9) below 70° for IPCI and below 80° for TFM. This can easily be explained by a greater part of the energy being reflected away from the path considered for the view. Moreover, this phenomenon was mainly observed for the T–T view, because, as the angle of the notch was increased, the energy was reflected away from the direct path to the array. This observation is accentuated for TFM as the reconstructed image is less sensitive to defect tips and is a better representation of the notch side, as shown in Figure 14, where this property is particularly notable for notch C.

## 4. Discussion

**Figure 15:**
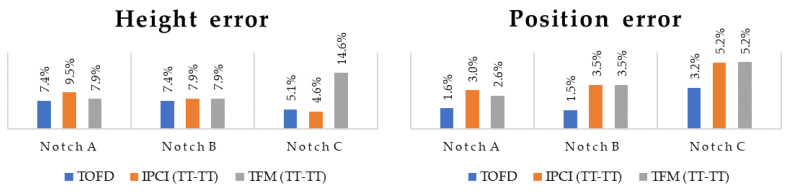
Comparison of height and position error between TOFD, IPCI TT–TT view, and TFM TT–TT view for notches A, B, and C.

When comparing both results obtained for TOFD and imaging techniques, it can be seen that the positioning error (below 7%) was smaller than the sizing error (below 10%). Thus, both the T–T and TT–TT views can be considered for characterization of notch-like defects if their angle respective to the horizontal plane remains above 80°. For higher angles, IPCI and the TT–TT view should be preferred, as they show a better accuracy for sizing (angles up to 70°) and a stable accuracy for localization (angles up to 60°). Angle limitation was however not observed for TOFD. A comparison of the TT–TT views in both TOFD and in the imaging techniques is summarized in Figure 15 for notches A, B, and C (T–T view and notch D presented too many discarded values). It can be seen that the results were very similar among all presented defects and metrics, although TOFD still has the better accuracy.

Beyond sizing and localization capabilities, imaging also allows a more comprehensive interpretation of inspection data, which is now fully accessible, as commercial phased array units are now capable of real-time TFM processing. As an example, while the inclination and length of defects were easily determined using TT–TT views, the orientation and length of notches, on the other hand, cannot be estimated using TOFD. While imaging techniques present some advantages over TOFD, there are also many advantages in using non-amplitude-based imaging methods. Thus, for the studied case, several benefits of IPCI over TOFD and TFM were identified:

- The inspection requires only one probe, reducing the footprint of the overall scanning setup as compared to TOFD. This allows the characterization of corner or tee joints. However, the orientation of the defect is critical for its detection, as incident energy has to be reflected back to the probe.
- Setting up the scanning is easier and less sensitive to the probe distance relative to the defect, unlike with TOFD.
- The image can be directly interpreted by the inspector and additional characteristics can be estimated, such as the defect orientation and exact location, avoiding uncertainties encountered with TOFD and the locus curve.
- Unlike with the TFM, the reconstructed image is not based on a signal amplitude, and therefore, no calibration is required for sizing, and the gain can be set to a very low value. Indeed, as can be observed in Figure 11, Figure 12, Figure 13 and Figure 14, the contrast is better on IPCI images relative to TFM ones.
- Sensitivity to defect tips, and more generally to diffractive geometries, is enhanced, and allows an accurate and easier sizing of the notch compared to TFM (Figure 14).

## 5. Conclusions

In this paper, TOFD was compared to instantaneous phase coherence imaging (IPCI) in terms of sizing and localization of crack-like defects. IPCI multi-view imaging was explored for the first time. The use of the TT–TT view showed characterization performances similar to those of TOFD. EDM notches machined in the center of the studied sample plate demonstrated that the angle of the defect plays an important role in the relevance of the IPCI method. Indeed, both the T–T and TT–TT views were shown to be usable for angles above 80° relative to the horizontal plane, while only the TT–TT gave satisfactory results for angles between 60° and 80°. On the other hand, TOFD showed relatively stable results among different notch angles. However, in the case of welds, reflectors are mostly located along the bevel, and it is highly unlikely to encounter angles below 70°. Conventional amplitude-based TFM images were also provided, and showed a similar behavior, while being less sensitive to defect tips.

IPCI presents a competitive advantage over TOFD, as it requires a single probe, is easier to set up and interpret, and allows the estimation of additional characteristics of the defect, such as orientation. It also has clear advantages over the amplitude TFM, as it is more sensitive to defect tips, making sizing easier, and image interpretation is not based on amplitude-calibrated signals. Finally, while imaging represents an easier approach for inspection interpretation, further work has to be done in order to reduce the number of acquired signals, the data size, and the acquisition time, in order to fully compete with TOFD.

## Figures and Tables

**Table 1:** Noise level sensitivity analysis results for a point like scatterer using both IPCI and conventional TFM algorithms where array performance indicator (API) was computed for each simulated configuration.

	SNR	Simulation 1	Simulation 2	Simulation 3
TFM	5 dB	0.54	0.93	1.35
	10 dB	0.54	0.94	1.35
	15 dB	0.54	0.94	1.35
	20 dB	0.54	0.94	1.35
IPCI	5 dB	0.89	1.37	1.77
	10 dB	1.04	1.66	2.22
	15 dB	1.45	1.93	2.6
	20 dB	1.78	2.37	2.96

**Table 2:** Experiment parameters.

Parameters	TOFD	IPCI
**Probe**		
Model	V564-SM	PWZ1
Central frequency	15 MHz	7.5 MHz
Elements	1	60
Elements pitch	-	1 mm
Elements width	∅ 3 mm	10 mm
**Wedge**		
Model	ST1-70L	SPWZ1-N55S
Angle (Refracted)	22° (70°)	36.1° (55°)
Material	Rexolite^®^	Rexolite^®^
**Acquisition**		
Voltage	295 V	50 V
Sampling frequency	100 MHz	62.5 MHz
PCS	69.8 mm	-
**Sample**		
Longitudinal velocity	5953 m/s	
Shear velocity	3243 m/s	

**Table 3:** Defect height, center position, and related errors using TOFD.

Defect	Height (mm)	Error Height (%)	Defect Center (mm)	Error Position (%)
A	5.37	7.4	9.66	1.6
B	5.37	7.4	9.64	1.5
C	5.26	5.1	9.81	3.2
D	5.24	4.7	9.66	1.7

**Table 4:** Defects height, center position, and related errors for both T–T and TT–TT views using the IPCI algorithm. Angle of the defect determined using the principal axis of the TT–TT view.

View	Defect	Height (mm)	Height Error	Defect Center (mm)	Position Error	Angle (°)
T–T	A (90°)	5.02	0.5%	10.04	6%	-
	B (80°)	4.77	4.7%	9.83	3%	-
	C (70°)	(2.67)	(46.5%)	9.28	2%	-
	D (60°)	(7.03)	(40.6%)	(11.30)	(19%)	-
TT–TT	A (90°)	4.52	9.5%	9.79	3%	93.6
	B (80°)	4.60	7.9%	9.83	3%	82.2
	C (70°)	4.77	4.6%	10.00	5%	72.7
	D (60°)	(3.79)	(24.2%)	9.99	5%	62.9

**Table 5:** Defects height, center position, and related errors for both T–T and TT–TT views using the TFM algorithm. Angle of the defect determined using the principal axis of the TT–TT view.

View	Defect	Height (mm)	Height Error	Defect Center (mm)	Position error	Angle (°)
T–T	A (90°)	4.85	3.0%	10.04	6%	-
	B (80°)	4.60	8.0%	9.91	4.3%	-
	C (70°)	(3.51)	(29.8%)	9.53	0%	-
	D (60°)	(7.11)	(42.2%)	(11.42)	(20%)	-
TT–TT	A (90°)	4.61	7.9%	9.75	3%	95.5
	B (80°)	4.60	7.9%	9.83	3%	84.03
	C (70°)	(4.27)	(14.6%)	10.00	5%	74.2
	D (60°)	(2.01)	(59.8%)	9.45	0%	61.1

## Data Availability

Data and codes used in this study are openly available at https://pulets.ca/open-data/sensors-21-00730.
